# Genetic diversity and differentiation characteristics of the widespread plant *Phragmites australis* in eastern China

**DOI:** 10.3389/fpls.2026.1995193

**Published:** 2026-10-29

**Authors:** Xiaokang Huo, Ningxu Li, Haoyang Yu, Chen Wang, Yajie Gao, Hongzhe Zhu, Yabin Wang, Lei Jin, Yajuan Zhang, Cunqi Liu

**Affiliations:** 1 College of Life Sciences, Hebei University, Baoding, China; 2 School of Biological Engineering, Aksu Vocational and Technical College, Aksu, China; 3 Monitoring & Management Room, Baoding Ecological and Environmental Monitoring Center, Baoding, China

**Keywords:** eastern China, genetic differentiation, genetic diversity, geographic pattern, *Phragmites australis*

## Abstract

**Introduction:**

*Phragmites australis* (common reed) is a globally distributed and highly productive emergent macrophyte with important ecological functions in wetland ecosystems. However, the genetic diversity and population differentiation of *P. australis* in eastern China remain insufficiently characterized.

**Methods:**

In this study, we performed RAD-seq-based reduced-representation genome sequencing of 98 *P. australis* individuals collected from populations distributed across eastern China to characterize genetic variation, population structure, and geographic differentiation among populations.

**Results:**

A total of 730,413 high-quality single-nucleotide polymorphisms (SNPs) were identified. Population genomic analyses revealed relatively high genetic diversity across eastern Chinese populations and substantial population structure, with the broad geographic distribution of *P. australis* associated with the formation of multiple genetic clusters. Functional enrichment analyses further indicated that pathways related to plant–pathogen interactions and sugar metabolism, highlighting potential biological processes associated with the genetic variation observed among the sampled populations. Among the sampled populations, the Longqi Bay (LQW) population exhibited the most distinct genetic characteristics, followed by the Baiyangdian Lake (BYD) and Xizhi Lake (XZH) populations. In contrast, the Weishan Lake (WSH) and Panjin (PJ) populations showed relatively low genetic differentiation and displayed genomic variation patterns more similar to those of the reference genome. Notably, the Yangtze River region coincided with a pronounced north–south transition in genetic structure.

**Discussion:**

Overall, these findings provide new insights into the population genetic structure, evolutionary history, and geographic differentiation of *P. australis* in eastern China and provide valuable genomic resources for investigating its evolutionary dynamics and ecological adaptation.

## 1 Introduction

*Phragmites australis* (Cav.) Trin. ex Steud., a perennial angiosperm belonging to the genus Phragmites within the Poaceae family, is one of the most widely distributed wetland plants worldwide. As a highly productive species, it plays important ecological roles in wetland ecosystems by contributing to primary production, nutrient cycling, habitat formation, and pollutant removal (Parde et al., 2021; Li et al., 2025). Due to its high tolerance to a wide range of environmental stresses and high biomass production, *P. australis* has also been widely applied in ecological restoration, including constructed wetlands for the removal of heavy metals and other contaminants from aquatic environments (Parde et al., 2021; Liu et al., 2025). In China, *P. australis* is also used in traditional medicine and various industrial applications, and its ecological functions, genetic diversity, and evolutionary characteristics have attracted increasing research attention in recent years (Ren et al., 2022; Shi et al., 2024).

Previous genetic and phylogeographic studies have revealed substantial genetic variation and geographic structuring in *P. australis*. A recent genome-wide RAD-seq study identified six major genetic lineages across the global distribution of the species and demonstrated that polyploidization, hybridization, and gene flow have contributed to its complex evolutionary history, with a distinct Chinese lineage and extensive genetic admixture among several lineages (Wang et al., 2024). Earlier chloroplast-based phylogeographic analyses identified multiple geographically distributed haplotypes and highlighted the influence of mutation types on inferred lineage relationships, while recent SSR-based research of Nanhui Dongtan in Shanghai further demonstrated genetic variation among Chinese reed populations (An et al., 2012; Ma et al., 2025). Phylogenetic studies in southwestern China have also identified differentiated lineages and their hybrids, suggesting that both geographic isolation and genetic exchange contribute to the diversification of *Phragmites* in China (Tanaka et al., 2017). Although populations from several major wetland regions in China, including the Yellow River Delta, Bohai Bay, the Minjiang River estuary, and the Yangtze River estuary, have been investigated from ecological and genetic perspectives (Liu et al., 2024; Zhang et al., 2024; Huang et al., 2025), systematic genome-wide analyses of population genetic variation across eastern China remain limited. In particular, the geographic patterns and potential environmental factors underlying genetic differentiation among *P. australis* populations across eastern China remain poorly understood.

*P. australis* exhibits a complex life-history strategy that may strongly influence its population genetic structure. As a perennial clonal plant, it can rapidly expand through extensive rhizome growth, allowing long-term persistence and dominance in wetland habitats. Meanwhile, *P. australis* is also capable of sexual reproduction through wind-pollinated flowers and produces numerous lightweight seeds that can be dispersed by wind, water, and animals (Meyerson and Cronin, 2013; Song et al., 2021). The coexistence of sexual reproduction and clonal propagation may generate complex genetic patterns, with local populations potentially maintaining high persistence while experiencing limited or variable levels of genetic diversity. In addition, environmental factors such as hydrological connectivity, habitat fragmentation, and geographical isolation may further regulate gene flow and genetic differentiation among populations. Therefore, understanding the genetic structure of *P. australis* requires consideration of both its reproductive strategy and ecological characteristics.

Eastern China contains extensive wetland habitats and supports widespread populations of *P. australis* across diverse geographic settings. Previous studies based on molecular markers have revealed multiple genetic lineages and complex population structure in *P. australis* at broader geographic scales. However, the patterns of genetic differentiation and population structure among geographically separated populations within eastern China remain insufficiently characterized. In particular, it remains unclear how geographic separation is associated with genetic differentiation at this regional scale. To address this knowledge gap, this study collected *P. australis* populations from ten representative regions in eastern China and employed RAD-seq-derived reduced-representation sequencing approach combined with reference genome alignment to characterize SNP-based genetic variation (Davey et al., 2011; Andrews et al., 2016). Specifically, this study aimed to investigate how geographic separation is associated with genetic differentiation and population structure of *P. australis* across eastern China. We hypothesized that geographic separation contributes to genetic differentiation among populations and predicted that geographically distant populations would tend to among exhibit greater genetic differentiation, consistent with a potential reduction in gene flow with increasing geographical distance. These findings contribute to a better understanding of the relationship between geographic separation and genetic differentiation in *P. australis* across eastern China.

## 2 Materials and methods

### 2.1 Field sample collection

Samples of *Phragmites australis* were collected from natural populations distributed across eastern China, covering eight provinces and one municipality directly under the central government (**Figure 1A**; **Supplementary Table 1**). Sampling sites were selected to represent the geographic distribution of *P. australis* in the study region. Within each population, individuals were randomly selected from the field sites, with relatively large spatial distances maintained between sampled individuals to minimize the possibility of repeatedly sampling individuals from the same vegetatively propagated clump. A total of 98 individuals were collected across ten populations distributed throughout eastern China (**Supplementary Table 1**). To minimize the influence of seasonal variation on sample quality and physiological status, young and healthy leaves were collected during the vegetative growth stage from May to September 2024. The collected leaf samples were immediately preserved in dry ice and transported to the laboratory for genomic DNA extraction.

**Figure 1:**
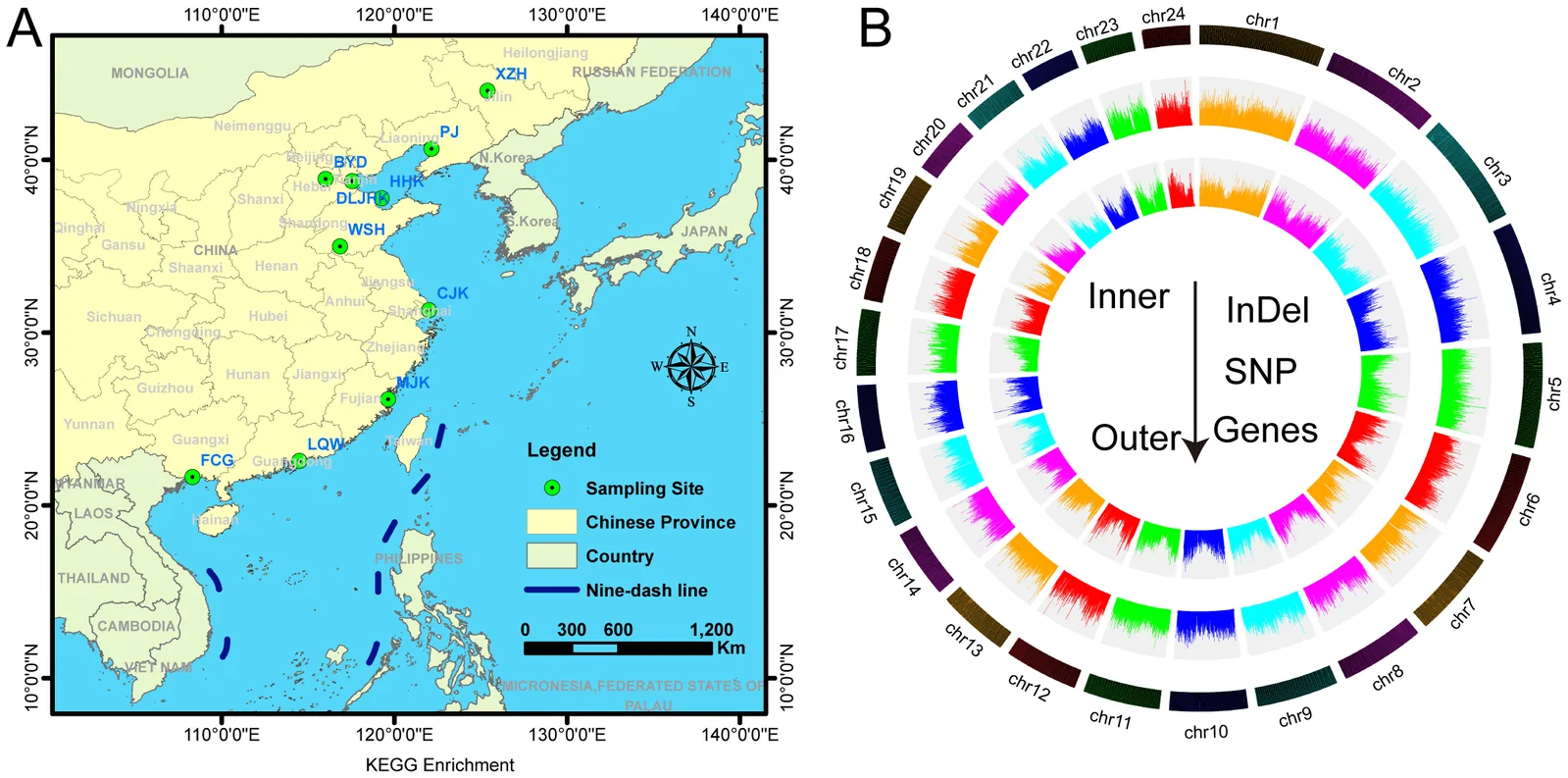
**(A)** Sampling point map. **(B)** Visualization of mutation information using a Circos plot, showing the distribution of genes, single-nucleotide polymorphisms (SNPs), and insertion–deletion variants (InDels) on the chromosomes from the outer to the inner layers. Bar height represents marker density at each genomic position. XZH, Xizhi Lake; PJ, Panjin; BYD, Baiyangdian Lake; DLJHK, Duliujian River estuary; HHK, Yellow River estuary; WSH, Weishan lake; CJK, Yangtze River estuary; MJK, MinJiang estuary; LQW, Longqi Bay; FCG, Fangchenggang.

### 2.2 Genomic DNA library preparation and sequencing

Genomic DNA was extracted from leaf tissues using the CTAB method. DNA quality was evaluated using two approaches: DNA integrity and potential contamination were examined by electrophoresis on 1% agarose gels, and DNA concentration was measured using an ND-2000 spectrophotometer (NanoDrop Technologies). Only DNA samples with high quality (OD_260/280_ = 1.8–2.0 and OD_260/230_ ≥ 2.0) were used for sequencing library construction.

For each sample, 0.5 μg of genomic DNA was used to construct RAD-seq libraries. Genomic DNA was digested with the restriction enzyme *EcoRI* to generate restriction-site-associated DNA fragments, followed by adapter ligation and sample multiplexing. The resulting DNA fragments were subjected to size selection, end repair, A-tailing, Y-adapter ligation, and PCR amplification. The amplified libraries were purified using the AMPure XP system, and fragment size distributions were evaluated using an Agilent 2100 Bioanalyzer. The expected insert size was approximately 330–480 bp, resulting in final library sizes of approximately 450–600 bp, including adapter sequences. Library concentrations were determined by real-time PCR.

Paired-end sequencing was performed on the Illumina NovaSeq X Plus platform at Shanghai Majorbio Bio-Pharm Technology Co., Ltd. Sequencing quality statistics, including raw reads, clean reads, and sequencing depth, are provided in **Supplementary Table 2**. The resulting genomic data were further used to assess genetic similarity among samples and to identify potential redundant individuals before downstream population genetic analyses.

### 2.3 Read processing, and variant calling

Raw sequencing reads were processed using Fastp v0.23.4 (Chen et al., 2018) to remove low-quality reads (mean Phred score < 20), adapter-contaminated sequences, and reads containing excessive ambiguous nucleotides (N bases > 10). After quality filtering, clean reads were retained for subsequent alignment and variant detection analyses.

*P. australis* has a complex genomic background associated with polyploidy, and the ploidy levels of individuals may vary among populations. Accurate assembly of polyploid plant genomes remains challenging. The chromosome-level *P. australis* reference genome GCF_958298935.1 used here is a collapsed assembly generated from an allotetraploid individual (Christenhusz et al., 2024), which was downloaded from the NCBI Genome database for read mapping. Constrained by reference genome limitations and uncertain ploidy of our samples, robust polyploid allele dosage estimation was not achievable. Variant calling was thus implemented within a diploid analytical framework. Notably, paralogous genomic regions were not explicitly filtered in our variant-calling pipeline. Sequence homology among paralogous loci may introduce bias to genotype inference, which represents a limitation of the present reference-based analysis.

Clean reads mapped to the reference genome of *P. australis* using BWA-MEM with default parameters (Jung and Han, 2022). The aligned BAM files were processed following the GATK Best Practices workflow (McKenna et al., 2010). Briefly, BAM files were sorted using Samtools (v1.15), and PCR duplicates were identified and removed using Picard MarkDuplicates (v2.26.10). Germline SNP and InDel variants were identified using Sentieon HaplotypeCaller and GVCFtyper (Freed et al., 2017) following a GVCF-based joint genotyping strategy. Variant calling was performed using the default diploid genotype model (ploidy = 2), without customized parameters for polyploid allele-dosage estimation. Accordingly, genotypes were represented using standard diploid genotype states (0/0, 0/1, and 1/1), and allele dosage was not estimated.

### 2.4 Variant quality filtering and population genetic SNP filtering

Both SNPs and InDels were identified during variant calling and were initially subjected to variant-level quality filtering. Variant quality filtering was performed based on the following criteria: QD < 2.0, FS > 30.0, SOR > 3.0, MQ < 40.0, MQRankSum < −3.0, ALT = “*”, or ReadPosRankSum < −3.0. The resulting high-quality polymorphic loci were subsequently subjected to additional population genetic filtering.

For downstream population genetic analyses, SNP loci were retained when the mean sequencing depth across individuals was ≥4× and ≤1000×, genotype missingness was <30% per locus, and the minor allele frequency (MAF) was ≥0.05. Multiallelic SNPs were excluded. Linkage disequilibrium (LD) pruning was subsequently performed using PLINK (v1.9) (Purcell et al., 2007) with the “--indep-pairwise 50 5 0.5” parameters. The resulting high-quality polymorphic SNP loci were used for genetic diversity estimation, population structure analysis, tree-based analysis of genetic relationships, and population differentiation analysis. No separate HWE filtering criterion or genotype-quality (GQ) threshold was applied.

### 2.5 Functional annotation and enrichment analysis

High-confidence variants were annotated using SnpEff (Cingolani et al., 2012) based on the genome annotation information of *P. australis*. SNPs and InDels were classified according to their genomic locations, including intergenic regions, exons, introns, splice sites, untranslated regions (UTRs), and 1-kb upstream and downstream regions. Functional effects of variants were categorized as synonymous mutations, missense mutations, start/stop codon alterations, and splice-site mutations. Genes associated with the annotated polymorphic loci were subsequently subjected to Gene Ontology (GO) and Kyoto Encyclopedia of Genes and Genomes (KEGG) annotation and enrichment analyses. Functional enrichment was used to uncover potential biological processes associated with inter-population genetic variation, offering mechanistic insights into population divergence.

### 2.6 Genetic diversity parameter

Heterozygosity reflects the level of genetic variation within a population and includes observed heterozygosity (*H_o_*) and expected heterozygosity (*H_e_*). *Ho* represents the proportion of heterozygous genotypes observed in a population, whereas *He* was calculated according to Nei’s gene diversity method (Nei, 1978), representing the probability that two randomly selected alleles are different. Nucleotide diversity (π) represents the average number of nucleotide differences per site between two randomly selected sequences (Yu et al., 2003), reflecting nucleotide-level genetic variation within populations. In a randomly mating population, π represents heterozygosity at the nucleotide level, reflecting the degree of polymorphism in population; the lower the polymorphism, the higher the level of selection.

The inbreeding coefficient (*F_IS_*) measures the deviation of observed heterozygosity from expected heterozygosity within a population due to inbreeding or population structure. The value of *F_IS_* ranges from −1 to 1, with negative values indicating an excess of heterozygotes and positive values indicating a deficiency of heterozygotes. An *F_IS_* value of zero indicates no deviation from expected heterozygosity.

Polymorphism information content (*PIC*) was used to evaluate the polymorphism and informativeness of genetic markers. Higher *PIC* values indicate greater marker polymorphism and allelic variation within populations. Shannon’s information index (*I*) was used to characterize allele diversity and allele frequency distribution at loci, with higher values indicating greater genetic diversity.

Population differentiation (*F_ST_*) measures the degree of genetic differentiation among populations and is influenced by evolutionary processes such as mutation, genetic drift, migration, selection, and population subdivision. The value of *F_ST_* generally ranges from 0 to 1, with values closer to 1 indicating stronger genetic differentiation among subpopulations. Following commonly used criteria, *F_ST_* values of 0–0.05, 0.05–0.15, and >0.15 represent low, moderate, and high genetic differentiation, respectively. Minor allele frequency (*MAF*) refers to the frequency of the less common allele at a polymorphic locus within a population.

The detailed formulas used for calculating genetic diversity parameters are provided in the Supplementary Materials and methods.

### 2.7 Geographic genetic differentiation analysis

The geographical genetic differentiation network was visualized using the Geo Layout plugin of Gephi (v0.10) and ArcGIS 10.8. To assess the isolation-by-distance (IBD) pattern among *P. australis* populations, pairwise Weir–Cockerham *F_ST_* was converted to Rousset’s linearized genetic differentiation metric, calculated as *F_ST_*/(1 − *F_ST_*) (Rousset, 1997). Geographic distances between the ten sampling sites were calculated from latitude and longitude coordinates using the “geosphere” package in R v4.4.2. Ordinary least-squares linear regression was used only to visualize the relationship between geographic distance and genetic differentiation. Statistical significance of the IBD relationship was assessed using a Mantel test with 10,000 permutations to account for the non-independence of pairwise distance matrices. Mantel tests were performed using the “ecodist” package in R v4.4.2. Simple linear regression lines shown in scatterplots were generated only for visualization and were not used for statistical inference. We acknowledge that Mantel tests may be sensitive to spatial autocorrelation, which may affect the statistical significance of the observed association between geographic distance and genetic differentiation. No partial Mantel test or other explicit correction for spatial autocorrelation was performed in the present study. Therefore, the IBD results were interpreted cautiously.

### 2.8 Phylogenetic analyses

Following LD pruning, the SNP-containing VCF file was converted into FASTA alignment format using vcf2phylip (v2.8). Only polymorphic SNP sites were retained, whereas invariant monomorphic sites were excluded. To reduce the influence of missing data, SNP sites with genotypes available for fewer than 90% of the sampled individuals were removed, resulting in a maximum missing-data proportion of 10% per site. Missing genotypes were encoded as N, and heterozygous genotypes were represented using the corresponding IUPAC ambiguity codes. The resulting SNP alignment was analyzed using FastTree 2.1.11 under the GTR+Gamma model with the approximate maximum-likelihood (aML) approach (Price et al., 2010). The resulting tree was used to provide a rapid visualization of genetic relationships and population structure among individuals and was interpreted as a complementary representation of SNP-based genetic differentiation. The resulting phylogenetic tree was subsequently visualized using the online platform iTOL (Letunic and Bork, 2024).

### 2.9 Population structure analysis

An unsupervised maximum-likelihood clustering algorithm implemented in ADMIXTURE (v1.3.0) was used to infer the genetic structure of the investigated populations (Alexander et al., 2009). ADMIXTURE analyses were conducted with the number of genetic clusters (*K*) ranging from 1 to 20 using default parameters. To visualize the genetic relationships among samples, we performed principal component analysis (PCA) based on pruned SNPs using Plink (v1.9) (Purcell et al., 2007). We used the Plink to estimate pairwise genetic relatedness among all individuals based on pairwise comparisons of SNP data (Manichaikul et al., 2010). The corresponding relatedness categories and kinship values are provided in **Supplementary Table 3**.

### 2.10 Linkage disequilibrium analysis

Genome-wide linkage disequilibrium (LD) decay was assessed by estimating the squared correlation coefficient (r²) between pairs of SNP loci using PopLDdecay (v3.42), based on RAD-seq-derived reduced-representation genome resequencing data (Zhang et al., 2019). The mean *r²* values were calculated across different physical distance intervals (0–1000 kb) between SNP pairs to characterize the LD decay pattern across the genome.

## 3 Results

### 3.1 Reduced-representation genome sequencing

In the reduced-representation genome sequencing analysis of 98 *P. australis* samples from eastern China, a total of 100.33 Gb of sequencing reads were generated, with a Q30 rate of 91.57% and a GC content of 46.52%. All samples passed quality control filtering (**Supplementary Table 2**). Following sequence clustering and variant calling, 55,054,643 tags, 13,429,377 SNPs, and 1,487,273 InDels were identified. After LD pruning, a total of 730,413 high-quality SNPs remained after the complete filtering procedure and were used for downstream population genetic analyses. SNPs and InDels were functionally annotated using SnpEff based on the *P. australis* genome annotation, and their genomic distributions and functional categories were further characterized. A circular genome map was constructed to visualize the genome-wide distribution of genes, SNPs, and InDels across chromosomes (**Figure 1B**). The outermost circle represents the 24 chromosomes, while the inner tracks display the distributions of annotated genes, SNPs, and InDels. The density of genetic variants varied among chromosomes, with several genomic regions exhibiting relatively high concentrations of SNPs and InDels, indicating heterogeneous patterns of sequence variation across the genome.

### 3.2 Functional enrichment analysis of polymorphic loci

To characterize the potential biological functions of genes containing polymorphic loci in *P. australis*, KEGG and GO enrichment analyses were performed, and significantly enriched pathways and functional categories were identified. The top 20 significantly enriched KEGG pathways are presented in **Figure 2A** and ranked according to −log10(*P*-value). Among these pathways, plant–pathogen interaction showed the highest Gene Ratio, followed by three sugar metabolism-related pathways, including starch and sucrose metabolism, glycolysis/gluconeogenesis, and amino sugar and nucleotide sugar metabolism. These pathways exhibited relatively high −log10(P-value) values, indicating significant enrichment of genes involved in these biological processes.

**Figure 2:**
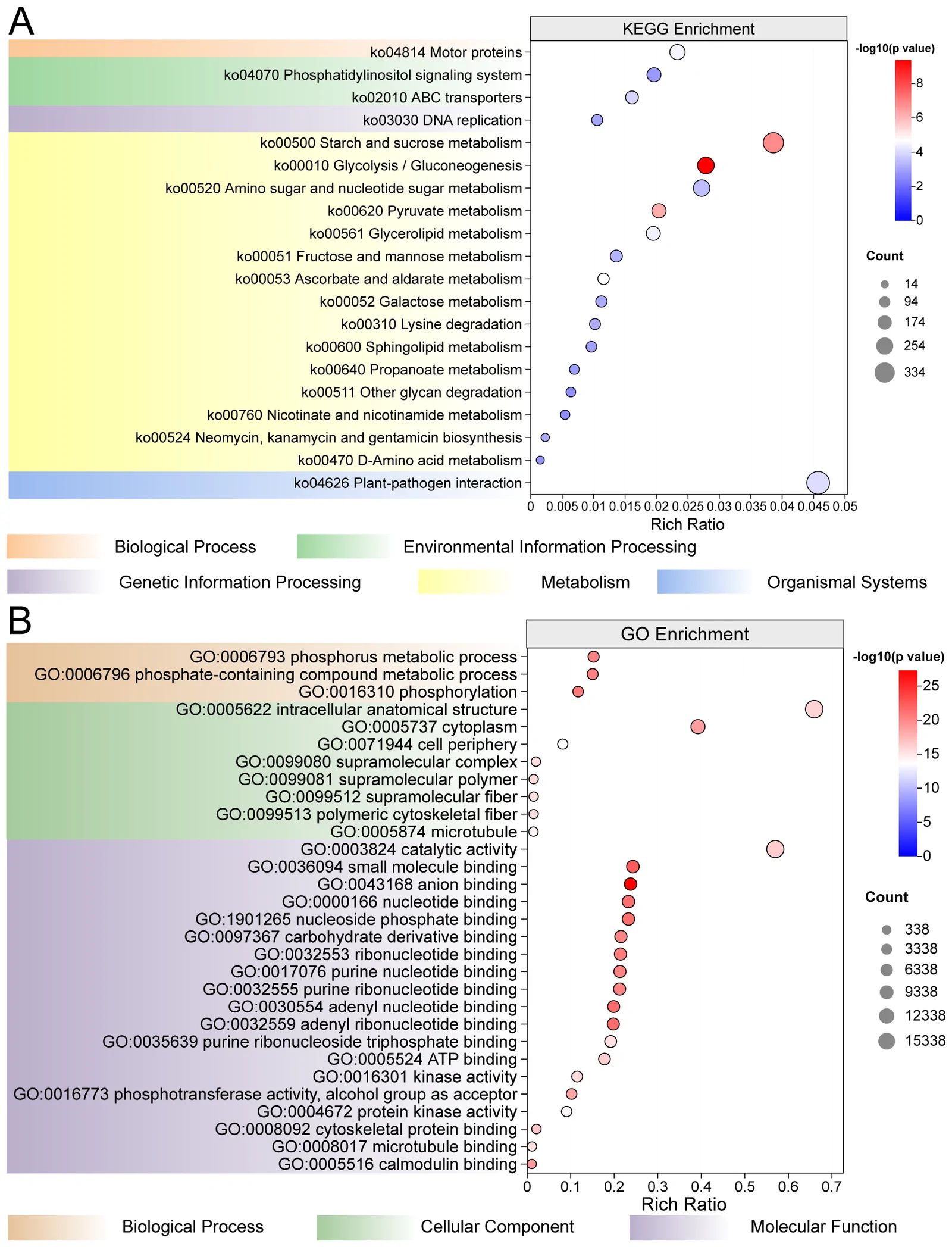
Analysis of the function of polymorphic loci. **(A)** KEGG enrichment analysis of variant detection results. **(B)** GO enrichment analysis of variant detection results. Gene Ratio: the ratio of the number of target genes enriched in a specific pathway to the total number of target genes; P adjust: adjusted P-value. Count: Frequency of occurrence in samples. b. Variant detection GO enrichment analysis chart.

In the GO functional enrichment analysis, enriched GO terms from three ontologies, including Molecular Function (MF), Cellular Component (CC), and Biological Process (BP), were identified based on the top 30 categories ranked by −log10(*P*-value) (**Figure 2B**). Among the MF categories, catalytic activity showed the strongest enrichment, followed by small molecule binding and anion binding. At the CC level, genes associated with genetic variants were significantly enriched in cytoplasmic components, with intracellular anatomical structure showing the highest Gene Ratio. In addition, supramolecular polymer, supramolecular fiber, and supramolecular complex were significantly enriched cellular components (*P*-value = 2.92 × 10^-^¹^6^), suggesting the involvement of genes related to macromolecular structural organization. For BP categories, phosphorus metabolism-related processes, including phosphorus metabolic process, phosphate-containing compound metabolic process, and phosphorylation, were significantly enriched among genes associated with genetic variation in *P. australis*. These processes exhibited relatively high Gene Ratios. Moreover, carbon metabolism-related biological processes, including carbohydrate metabolic process and organic substance catabolic process, were also significantly enriched. Collectively, these enriched functional categories hint at potential biological processes that may be linked to the genetic differentiation among sampled P. australis populations.

### 3.3 Genetic diversity analysis

To assess the genetic diversity of *P. australis*, intra-population diversity parameters and population differentiation were calculated based on filtered population SNPs (**Table 1**; **Figure 3A**). The results indicated that the FCG population in Guangxi Province possessed the highest number of private SNPs, whereas the WSH and PJ populations exhibited the fewest. The genetic diversity results of *P. australis* populations in ten *P. australis* populations showed that the LQW area had the lowest levels of *H_o_*, *H_e_*, *PIC*, *I*, and *MAF*, followed by BYD and XZH. All ten populations yielded negative *F_IS_* values (ranging from −0.225 to −0.015), consistent with the observation that observed heterozygosity (*H_o_*) was universally higher than expected heterozygosity (*H_e_*). Among the ten regions, LQW was found to have the lowest *H_o_* and *H_e_*, at 0.04 and 0.028, respectively. CJK had the highest *H_o_* value at 0.336. The *H_e_* was highest in PJ and WSH, both at 0.24, and the *PIC* and *I* indices of PJ and WSH were also the highest among the ten regions, at 0.196 and 0.37, respectively. MAF showed that CJK was the highest (0.179). Except for LQW, the MAF values of coastal *P. australis* populations were relatively close, ranging from 0.168 to 0.179, higher than those of inland BYD and XZH.

**Table 1:** Genetic diversity index of ten populations.

Pop ID	Private	Num_Indv	*H_o_*	*H_e_*	*F_IS_*	*PIC*	*I*	*MAF*
BYD	486	8.839	0.157	0.123	-0.050	0.097	0.181	0.095
CJK	411	9.012	0.336	0.228	-0.190	0.180	0.337	0.179
DLJHK	471	8.532	0.329	0.230	-0.174	0.183	0.340	0.177
FCG	1409	8.703	0.325	0.197	-0.225	0.152	0.281	0.168
HHK	344	8.824	0.304	0.216	-0.140	0.170	0.316	0.170
LQW	810	5.107	0.040	0.028	-0.015	0.022	0.041	0.022
MJK	463	8.701	0.315	0.221	-0.165	0.176	0.327	0.171
PJ	66	8.662	0.303	0.240	-0.100	0.196	0.370	0.173
WSH	27	8.583	0.303	0.240	-0.100	0.196	0.370	0.172
XZH	199	7.220	0.201	0.154	-0.053	0.126	0.241	0.115
Average	468.6	8.218	0.261	0.1877	-0.121	0.149	0.280	0.144

Private, number of SNPs unique to the population. Num_Indv, average number of individuals successfully genotyped for each locus in each population. *H_o_*, observed heterozygosity; *H_e_*, expected heterozygosity; *F_IS_*, inbreeding coefficient; *PIC*, polymorphism information content; *I*, Shannon’s information index; *MAF*, minor allele frequency; XZH, Xizhi Lake; PJ, Panjin; BYD, Baiyangdian Lake; DLJHK, Duliujian River estuary; HHK, Yellow River estuary; WSH, Weishan lake; CJK, Yangtze River estuary; MJK, MinJiang estuary; LQW, Longqi Bay; FCG, fangchenggang.

**Figure 3:**
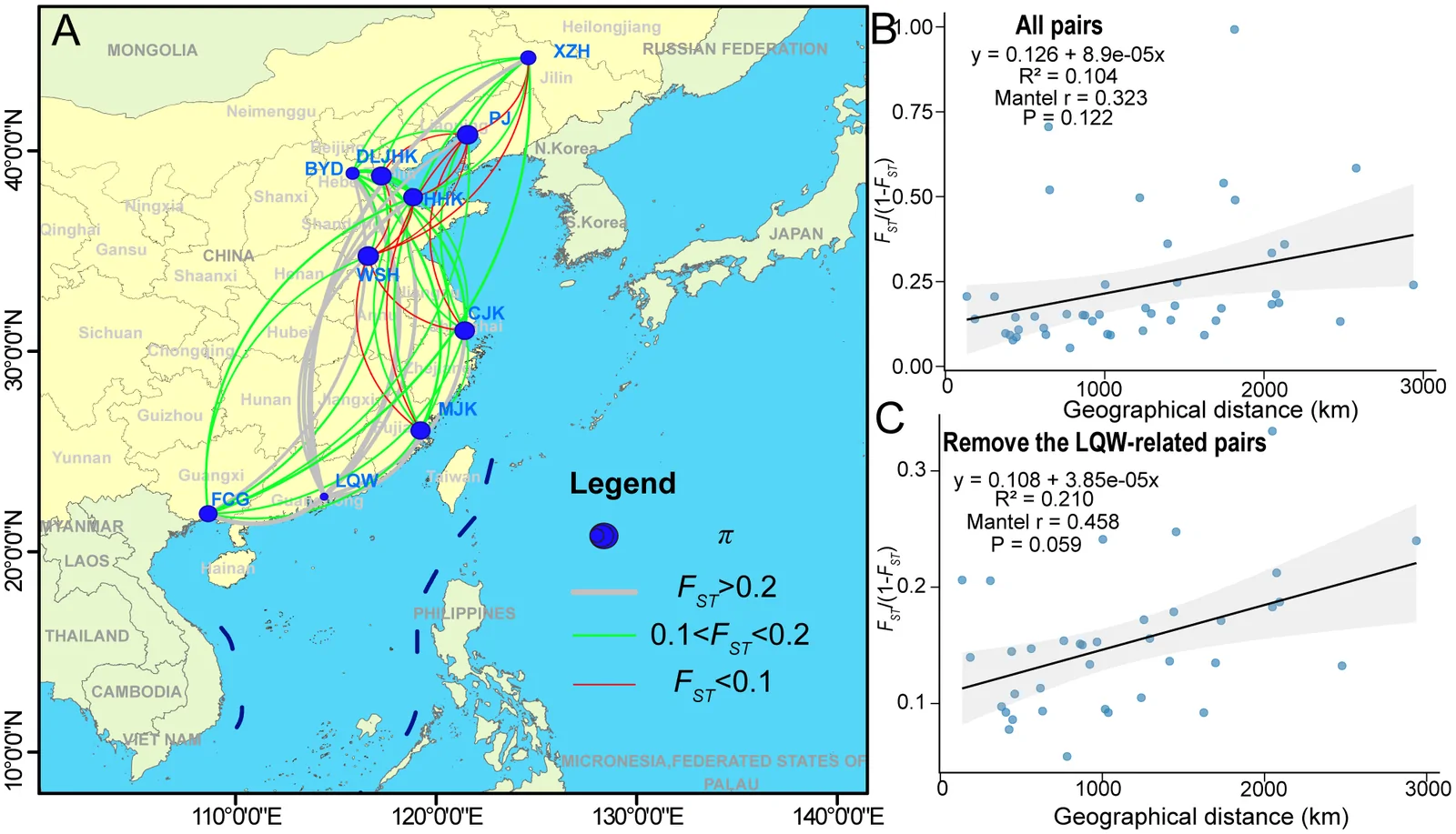
Sampling locations, nucleotide polymorphism and isolation-by-distance patterns of Phragmites australis populations across eastern China. **(A)** Geographic distribution map of nucleotide polymorphism and population differentiation (*F_ST_*). Circle size indicates the relative magnitude of the nucleotide polymorphism (π), and line width and reflects the relative magnitude of *F_ST_*. **(B)** Isolation-by-distance plot showing the relationship between Rousset’s linearized genetic distance *F_ST_*/(1- *F_ST_*) and pairwise geographical distance for all population pairs. The black solid line represents linear regression fit for visualization; Mantel test statistics are given in the upper-left corner. **(C)** Isolation-by-distance plot after removing all pairwise comparisons involving the LQW population. The black solid line represents linear regression fit for visualization; Mantel test statistics are given in the upper-left corner. XZH, Xizhi Lake; PJ, Panjin; BYD, Baiyangdian Lake; DLJHK, Duliujian River estuary; HHK, Yellow River estuary; WSH, Weishan lake; CJK, Yangtze River estuary; MJK, MinJiang estuary; LQW, Longqi Bay; FCG, fangchenggang.

A geographically based network map was used to display the relative magnitude of the nucleotide polymorphism (π), with connections among geographic populations represented by *F_ST_* values (**Figure 3A**). The results indicated that, except for LQW (π = 0.0325), nucleotide polymorphism (π) values ranged from 0.131 to 0.256. The highest π values were observed in the PJ and WSH populations. Moreover, genetic differentiation (*F_ST_*) between these two populations was the lowest (*F_ST_* = 0.052). LQW and other regions showed high levels of *F_ST_* (*F_ST_*> 0.2). The genetic similarity of *P. australis* in the coastal waters to the northeast and Weishan Lake is relatively high. Although Baiyangdian Lake and the northern *P. australis* are geographically close, the genetic distance is relatively far (*F_ST_* > 0.1). The populations with the highest *F_ST_* were BYD and LQW (*F_ST_* = 0.498). Isolation-by-distance (IBD) analysis was performed using Rousset’s linearized genetic distance against pairwise geographical distance. When all population pairs were included, genetic differentiation showed a weak positive but non-significant correlation with geographical distance (Mantel*r* = 0.323, *P* = 0.122). After removing all LQW-related pairwise comparisons, both the correlation strength and the marginal statistical signal of the fitted IBD relationship were markedly improved, yielding a moderate-to-strong positive correlation with marginally significant support for IBD (Mantel *r*= 0.458, *P* = 0.059). (**Figure 3B**).

### 3.4 Analysis of population genetic relationship and structure

The approximate maximum-likelihood (aML) tree based on the filtered RAD-seq-derived SNP dataset revealed clear genetic differentiation among the sampled *P. australis* populations in eastern China (**Figure 4A**). Individuals from the northern populations, including BYD, XZH, HHK, PJ, WSH, and DLJHK, generally clustered together, whereas individuals from the southern populations, including CJK, FCG, and MJK, formed separate groups. LQW was positioned separately from the major northern and southern groups, indicating a distinct genetic composition. Overall, the tree revealed a pronounced north–south pattern in the genetic relationships among the sampled populations.

**Figure 4:**
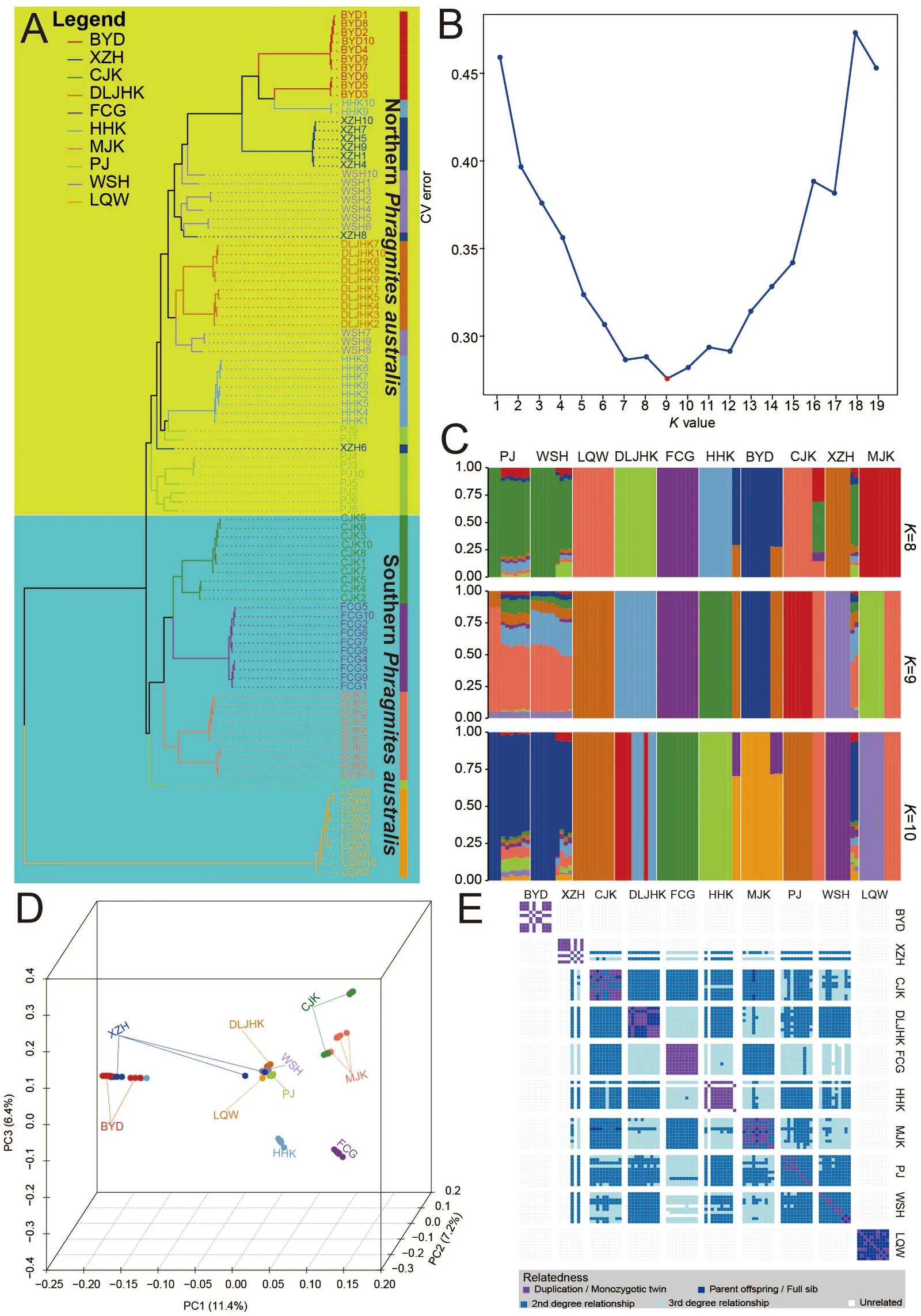
Population genetic relationships and structure of Phragmites australis populations across eastern China. **(A)** Approximate maximum-likelihood phylogenetic tree for all individual samples. **(B)** Cross-validation error plot for ADMIXTURE analysis across different *K* values. The x-axis shows putative genetic cluster numbers (K), and the y-axis corresponds to cross-validation error. The red point marks the K value with the lowest cross-validation error. **(C)** Representative ADMIXTURE genetic composition plots for *K* = 8, 9, and 10. Each color represents a genetic component, and each row shows the assignment of individuals to groups. **(D)** Three-dimensional principal-component analysis (3D-PCA) of individual genotypes. Each dot corresponds to one sample, colored according to its sampling locality. Genetically similar individuals tend to cluster together. **(E)** Pairwise sample kinship matrix. Each cell corresponds to a sample pair, with color intensity indicating the magnitude of pairwise kinship coefficients. XZH, Xizhi Lake; PJ, Panjin; BYD, Baiyangdian Lake; DLJHK, Duliujian River estuary; HHK, Yellow River estuary; WSH, Weishan lake; CJK, Yangtze River estuary; MJK, MinJiang estuary; LQW, Longqi Bay; FCG, fangchenggang.

To further characterize population genetic structure, we performed principal-component analysis (PCA) and pairwise kinship analysis. The three-dimensional PCA plot (**Figure 4D**) was consistent with the phylogenetic patterns. PC1, PC2 and PC3 explained 11.4 %, 7.2 % and 6.4 % of total genetic variance, respectively (**Supplementary Figure 1**). Individuals of northern and southern populations largely separated along the principal components, while LQW formed a discrete cluster, in agreement with the aML tree topology. Pairwise kinship analysis was implemented to evaluate relatedness among individuals across all ten populations (**Figure 4E**; **Supplementary Table 3**). Most pairwise comparisons fell into the unrelated or distant-related categories. The FCG population exhibited abundant within-population close kinship signals, with numerous individual pairs yielding high kinship values according to the thresholds defined in **Supplementary Table 3**. By contrast, BYD, PJ and WSH displayed relatively weak internal kinship, dominated by unrelated individual pairs. Across inter-population comparisons, BYD, XZH and LQW showed distant genetic relatedness relative to other populations. Several within-population individual pairs across the remaining populations also exhibited moderate-to-low kinship corresponding to second- or third-degree relatedness, as classified by **Supplementary Table 3**.

Population genetic structure was further evaluated using ADMIXTURE. The CV error decreased from *K* = 2 and reached its minimum at *K* = 9, after which it increased, indicating that *K* = 9 had the lowest CV error among the tested models (**Figure 4B**). The inferred genetic compositions across *K* = 2–19 are presented in **Supplementary Figure 2**, while representative results for *K* = 8–10 are shown in **Figure 4C**. Consistent with the pattern revealed by principal-component (PCA) analysis, ADMIXTURE also revealed clear genetic divergence between northern and southern populations, alongside the genetically distinct LQW population. At *K* = 8–10, the sampled individuals exhibited complex combinations of multiple genetic clusters, with substantial differences in genetic composition among populations. PJ and WSH showed relatively similar genetic compositions, whereas XZH contained individuals with genetic components resembling those observed in PJ and WSH. The southern populations, particularly CJK, FCG, and MJK, generally exhibited genetic compositions distinct from those of the northern populations. Overall, the ADMIXTURE results indicated substantial genetic structure and varying degrees of genetic admixture among *P. australis* populations in eastern China.

### 3.5 Population genetic linkage disequilibrium analysis

Linkage disequilibrium (LD) decay patterns were analyzed to evaluate the extent of linkage among SNP markers across different populations (**Supplementary Figure 3**). The LD levels (*r²*) of all populations exhibited a gradual decline with increasing physical distance between SNP loci, demonstrating a consistent pattern of linkage disequilibrium decay across populations.

However, substantial differences in LD decay rates were observed among populations. Among the analyzed populations, BYD, XZH, FCG, and HHK exhibited relatively slower LD decay, maintaining higher *r²* values at larger physical distances. In contrast, PJ and WSH showed a much faster decline in LD, with *r²* values decreasing rapidly within the first several hundred kilobases. The remaining populations, including CJK, DLJHK, MJK, and LQW, displayed intermediate LD decay patterns.

The quantitative parameters of LD decay were summarized in **Supplementary Table 4**. The half-decay distance varied considerably among populations, ranging from 167.898 kb in PJ to 1000 kb in BYD, XZH, FCG, and HHK. Similarly, the distance at which LD decreased to an *r²* value of 0.1 (decay01) ranged from 606.936 kb in PJ to approximately 1000 kb in several populations. PJ and WSH exhibited the shortest LD decay distances, with half-decay values of 167.898 kb and 183.522 kb, respectively, whereas multiple populations showed substantially longer LD persistence.

## 4 Discussion

### 4.1 Distribution pattern and functional characteristics of *P. australis* in eastern China

Our population genomic analyses revealed pronounced geographic variation in the genetic structure of *P. australis* across eastern China. *P. australis* is widely distributed worldwide and exhibits considerable genetic and phenotypic variation across different regions. Due to its strong environmental adaptability and rapid colonization ability, it has received extensive attention in ecological and evolutionary studies. In eastern China, where wetland habitats are widely distributed, large populations of *P. australis* occur; however, comprehensive investigations of its population genetic differentiation remain limited.

Both the 3D-PCA and sample kinship analyses revealed that the LQW population located in Shenzhen exhibited a distinct genetic pattern compared with other populations, accompanied by relatively lower genetic diversity indices. Given the intensive human activities in this region, historical population introduction or human-mediated dispersal may have contributed to this pattern. Nevertheless, further sampling and demographic analyses are needed to test this hypothesis. Furthermore, the BYD and XZH populations exhibited unique patterns of genetic differentiation, characterized by population-specific genetic variation. BYD located in the Xiong’an New Area of China and constitutes the largest freshwater wetland in North China, often referred to as the ‘Kidney of North China’. The *P. australis* wetlands in this area are widely recognized as sinks for intercepting and absorbing pollutants (Gao et al., 2013; Liao et al., 2024). The distinct genetic characteristics observed in BYD may reflect the influence of its geographic isolation, local population history, or environmental conditions. Similarly, the differentiation observed in XZH suggests that local demographic processes may have contributed to population divergence. However, the absence of corresponding environmental data prevents direct assessment of the contribution of habitat-specific selection to these genetic patterns.

The remaining populations, including PJ, CJK, HHK, MJK, and WSH, showed different levels of genetic variation and differentiation. The PJ population was collected from the Liao River estuarine wetland, one of the important wetland systems in northern China. Previous studies have highlighted the ecological importance and extensive distribution of *P. australis* in this region (Wang and Wei, 2012). The relatively large and continuous wetland habitat may provide opportunities for population persistence and gene exchange, which could contribute to the observed genetic patterns. CJK, HHK, and MJK populations were collected from estuarine wetlands along the eastern coast of China, where *P. australis* populations are widely distributed (Huang et al., 2017; Liu et al., 2021; Wu et al., 2024). The geographic distribution and connectivity of these coastal wetlands may influence historical dispersal and gene flow among populations. The WSH population was sampled from Weishan Lake, the largest meadow-type lacustrine wetland in Asia. Previous investigations have documented the extensive distribution of *P. australis* in this region, although population genetic information remains limited. The genetic characteristics observed in WSH may therefore provide new insights into the evolutionary history of lacustrine *P. australis* populations.

These populations are distributed across diverse wetland systems in eastern China, representing different geographic and ecological contexts. Their genetic patterns likely reflect the combined effects of historical dispersal, population connectivity, and local environmental conditions. Future studies integrating broader geographic sampling, ecological measurements, and environmental association analyses will be necessary to further clarify the drivers shaping genetic differentiation in *P. australis* populations.

### 4.2 Potential functional mechanisms associated with genetic variation and population differentiation

Functional annotation of polymorphic loci associated with population differentiation was performed by comparison with the reference genome. The enrichment of the plant–pathogen interaction pathway suggests a potential role of pathogen-related defense mechanisms in *P. australis* populations. Previous studies have shown that *P. australis* interacts with diverse microbial pathogens and that different lineages exhibit variation in pathogen susceptibility, indicating that host genetic background may influence plant–pathogen interactions (Nelson and Karp, 2013; Crocker et al., 2015; Allen et al., 2020). About the enrichment of three sugar metabolism-related pathways. Previous studies have shown that sucrose synthesis, transport, and utilization are closely associated with carbon allocation and rhizome development in *P. australis*, supporting its perennial growth strategy (Cui et al., 2025). In addition, changes in soluble sugars, starch accumulation, and glycolytic activity have been observed under salinity and other environmental conditions, highlighting the importance of carbohydrate metabolism in osmotic regulation and plant physiological responses (Hartzendorf and Rolletschek, 2001; Nada et al., 2015). Therefore, the enrichment of pathogen-related and sugar metabolism-related pathways may reflect biological processes associated with the genetic variation and population differentiation observed in *P. australis*.

GO enrichment analysis further revealed that genes associated with genetic variation in *P. australis* were significantly enriched in biological processes related to phosphorus metabolism, carbon metabolism, and catalytic activity. Phosphorus metabolism is essential for plant growth and development, as phosphorus participates in energy metabolism, signal transduction, and the regulation of nutrient homeostasis. Plants have evolved complex regulatory mechanisms involving phosphate sensing, transport, and utilization to cope with fluctuations in phosphorus availability (López-Arredondo et al., 2014; Satheesh et al., 2022). Therefore, the enrichment of phosphorus metabolism-related genes suggests that variation in nutrient-related processes may contribute to genetic differentiation among *P. australis* populations. In addition, carbon metabolism-related processes, including carbohydrate metabolism and organic substance catabolism, were enriched among genes associated with population variation. Carbohydrates not only provide energy for plant growth but also function as signaling molecules involved in plant development and responses to environmental changes (Devi and Kumar, 2024; Pang et al., 2024). Collectively, these enriched biological processes may represent potential functional mechanisms associated with the observed genetic variation and population differentiation in *P. australis*.

It should be noted that functional enrichment based on polymorphic loci does not identify genes under selection or establish a direct link between genetic variation and environmental adaptation. Therefore, the enriched pathways and biological processes identified here should be interpreted as candidate processes associated with population differentiation rather than evidence of adaptive evolution or habitat-specific selection. Future studies integrating environmental variables, genotype–environment association analyses, and selection scans will be necessary to evaluate the potential adaptive significance of these candidate biological processes.

### 4.3 Spatial patterns of genetic diversity and differentiation

The genetic diversity indices indicated that the *H*_o_, *H*_e_, *PIC*, *I*, *MAF*, and π values of PJ were relatively high. Panjin Wetland, one of the largest coastal *P. australis* marsh ecosystems worldwide, is characterized by extensive and relatively continuous habitats with limited fragmentation (Cheng et al., 2023). Such habitat continuity may maintain large effective population sizes and facilitate gene flow, thereby reducing genetic variation loss caused by genetic drift. In addition, the coexistence of freshwater, brackish, and saline marsh habitats creates substantial environmental heterogeneity, which may provide diverse ecological niches and contribute to the maintenance of genetic variation through potential local adaptation. Previous studies have shown that *P. australis* can adapt to salinity stress through multiple physiological mechanisms, including sodium exclusion, vacuolar sequestration, osmotic regulation, and antioxidant activation (Eller et al., 2017). Therefore, the high genetic diversity of PJ populations may reflect the combined effects of continuous habitat structure, environmental heterogeneity, and population connectivity.

The observed heterozygosity was generally higher than the expected heterozygosity across populations, resulting in negative *F*_IS_ values. This heterozygote excess may reflect multiple biological and methodological factors. As a perennial species capable of both sexual and vegetative reproduction, *P. australis* may maintain relatively high heterozygosity through outcrossing and gene flow, while clonal propagation may also influence heterozygosity estimates (Clevering and Lissner, 1999; Lambertini et al., 2008). In the present study, relatively large spatial distances were maintained between sampled individuals to minimize repeated sampling from the same vegetatively propagated clump, and kinship analysis was used to characterize genetic relatedness among individuals. Nevertheless, the contribution of clonality cannot be completely excluded.

In addition, the complex polyploid genomic background of *P. australis* and the use of a diploid reference genome and diploid genotype model may introduce uncertainty into heterozygosity and *F*_IS_ estimates. Allele dosage was not estimated, and paralogous regions were not specifically filtered. Moreover, SNP ascertainment in reduced-representation sequencing may influence allele-frequency distributions and downstream population genetic estimates (Nielsen and Signorovitch, 2003; Lachance and Tishkoff, 2013). These limitations should be considered when interpreting the negative *F*_IS_ values. Future studies incorporating ploidy-aware genotyping, allele-dosage estimation, more comprehensive genomic sampling, and explicit analyses of clonality and mating systems would help clarify the biological and methodological factors underlying the observed heterozygote excess.

Given the long coastline and extensive inland distribution of *P. australis* in China, populations are exposed to broad salinity gradients. Although *P. australis* exhibits considerable salt tolerance, elevated salinity can negatively affect biomass production, plant height, stem density, and rhizome carbohydrate accumulation (Achenbach and Brix, 2014; Ding et al., 2023). Therefore, populations occurring among freshwater-to-saline gradients may represent different evolutionary trajectories shaped by both environmental filtering and historical population processes. The unique genetic characteristics observed in BYD may therefore result from a combination of its distinct freshwater marsh environment, limited historical connectivity, and potential demographic isolation.

Moreover, the genetic structure and phylogenetic relationships over region coinciding with a pronounced transition in genetic structure. Previous studies have shown that the spatial genetic structure of *P. australis* can be associated with geographic distribution, environmental conditions, and historical colonization processes (Lambertini et al., 2008; An et al., 2012; Guo et al., 2018). More generally, geographic distance and landscape characteristics can influence gene flow and population connectivity in plants, while habitat fragmentation may increase spatial isolation and genetic differentiation among populations (Young et al., 1996; Holderegger et al., 2010). In the present study, differences in temperature, precipitation, light availability, and hydrological conditions across latitudes may contribute to environmental heterogeneity among populations. However, these factors should be considered potential contributors rather than exclusive determinants of the observed genetic variation. The observed north–south genetic pattern may therefore reflect the combined influence of isolation by distance, environmental heterogeneity, population connectivity, historical colonization, historical gene flow, and other demographic processes. Therefore, the Yangtze River region is better interpreted as a geographic transition zone associated with genetic structure rather than as a demonstrated physical barrier to gene flow. Further studies integrating environmental variables, historical demographic inference, and landscape genetic approaches will be necessary to disentangle the relative contributions of these factors to the observed population genetic structure.

Linkage disequilibrium (LD) decay reflects the extent of non-random association among genetic variants and can be influenced by multiple factors, including effective population size, recombination rate, mating system, demographic history, gene flow, population structure, and sampling design (Pritchard and Przeworski, 2001; Slatkin, 2008). In reduced-representation sequencing data, marker density and genomic distribution may also affect estimates of LD decay. In the present study, the relatively rapid LD decay observed in PJ and WSH indicates lower levels of LD among the sampled individuals with increasing physical distance, but should not be interpreted directly as evidence of higher genetic diversity or stronger population connectivity. Differences in LD decay among populations, including BYD and XZH, may reflect the combined effects of population history, gene flow, population structure, recombination, and sampling-related factors. Thus, the observed LD patterns should be interpreted as population-specific genetic patterns rather than evidence of a single underlying mechanism. Further studies integrating demographic inference and more comprehensive genomic sampling will be needed to clarify the factors contributing to variation in LD among *P. australis* populations.

### 4.4 Complex population genetic structure of *P. australis* as a widespread species

ADMIXTURE analysis based on the optimal *K* value revealed nine genetic clusters among the ten sampled *P. australis* populations. The wide geographic distribution may contribute to the formation and maintenance of genetic differentiation by exposing populations to diverse environmental conditions and increasing the possibility of spatial genetic structuring. Similar patterns have been observed in other widely distributed plant species. For example, genome-wide association study of 212 maize inbred lines identified the lowest cross-validation error at *K* = 15 (Qu et al., 2022), and population genetic analysis of chickpea revealed a complex genetic structure with the optimal *K* value of 13 (S et al., 2025). Although these species differ in evolutionary history and domestication background, their broad geographic distributions are associated with extensive genetic variation and population differentiation. These examples support the hypothesis that widely distributed species may harbor complex genetic structures due to long-term adaptation across heterogeneous environments and geographically separated populations.

In this study, *P. australis* exhibited substantial genetic variation among the sampled populations, with genetic diversity indices showing relatively high levels. As a widely distributed wetland species, *P. australis* occupies diverse habitats ranging from freshwater marshes to saline coastal wetlands, which may promote the accumulation and maintenance of genetic variation. The high genetic diversity and complex genetic structure observed in eastern Chinese populations may provide important genetic resources for understanding environmental adaptation and enhancing ecological resilience under future climate change scenarios. Nevertheless, the genetic diversity characterized in this study represents only a regional component of the species-wide variation, and broader geographic sampling will be required to further resolve the global genetic structure of *P. australis*.

## 5 Conclusions

For *Phragmites australis* populations collected from eastern China, 100.33 Gb of high-quality sequencing data were generated, identifying 13,429,377 SNPs and 1,487,273 InDels. Functional enrichment analysis identified plant–pathogen interaction and carbohydrate metabolism as potential biological processes associated with the genetic variation and population differentiation observed among populations. Population genetic analyses revealed significant differentiation among geographic populations, with LQW, BYD, and XZH populations showing distinct genetic structures. PJ and WSH shared the highest values of *H*_e_, *PIC*, and Shannon index, whereas CJK showed the highest *H*_o_ and *MAF* values. A pronounced north–south transition in genetic structure was observed along the latitudinal gradient, with the Yangtze River region coinciding with this transition. Nine genetic clusters were detected across ten populations, reflecting the genetic diversity and complex evolutionary history of widespread species characteristics of *P. australis*. This study provides new insights into the spatial genetic structure and genetic differentiation of *P. australis* populations and provides genomic resources that may support future studies of its evolutionary history, population connectivity, and conservation and management of wetland populations.

## Data Availability

The raw sequence data reported in this paper have been deposited in the Genome Sequence Archive in National Genomics Data Center, China National Center for Bioinformation/Beijing Institute of Genomics, Chinese Academy of Sciences (GSA: CRA036751) under project accession number PRJCA055356 which are publicly accessible at https://ngdc.cncb.ac.cn/gsa.
