# Supplementary material for: Genetic diversity and differentiation characteristics of the widespread plant Phragmites australis in eastern China
Source: Front Plant Sci. 2026 Oct 29;17:1995193. doi: 10.3389/fpls.2026.1995193 (PMC13755207; doi:10.3389/fpls.2026.1995193)
Supplement: Supplementary file 1 [file DataSheet1.docx]

Supplementary Material

# 1 Supplementary Materials and methods

Genetic diversity parameter

The formula genetic diversity parameters are as follows:

$$H_{o}=\frac{\sum_{l=1}^{m} n_{l}}{\sum_{l=1}^{m} t_{l}}$$

*m*: number of loci, $n_{l}$: number of heterozygous genotypes at the l-th locus, $t_{l}$: the number of individuals at the l-th locus.

$$H_{e}=1-\frac{1}{m}\sum_{l=1}^{m} \sum_{i=1}^{k} P_{li}^{2}$$

*m*: the number of loci, *k*: the total number of alleles at the l-th locus, 𝑃_𝑙𝑖_: the gene frequency of the *i*-th allele at the *l*-th locus.

$$\pi=\sum_{ij}^{q} \hat{x_{\text{i}}}\hat{x_{\text{j}}}d_{ij}$$

$q$: the total number of alleles, $\hat{x_{\text{i}}}$ and $\hat{x_{\text{j}}}$: The population frequency of the *i*-th and *j*-th alleles, $d_{ij}$: the number of nucleotide differences or substitutions per site

between the *i*-th and *j*-th alleles.

$$F_{is}=\frac{H_{e}-H_{o}}{H_{e}}$$

$$PIC=1-\sum_{i=1}^{n} p_{i}^{2}-\sum_{i=1}^{n} \sum_{j=i+1}^{n} 2p_{i}^{2}p_{j}^{2}$$

$$I=-\sum_{i=1}^{n} p_{i}\cdot ln(p_{i})$$

$p_{i}$ and $p_{j}$: the frequencies of the *i*-th and *j*-th alleles. 𝑛: number of different alleles at that locus.

$$\hat{\theta}=\frac{s^{2}-[\overline{p}(1-\overline{p})-\frac{r-1}{r}s^{2}]\frac{1}{2\overline{n}-1}}{[1-\frac{2\overline{n}C^{2}}{(2\overline{n}-1)r}]\overline{p}(1-\overline{p})+[1+\frac{2\overline{n}(r-1)C^{2}}{（2\overline{n}-1）r}]\frac{s^{2}}{r}}$$

$\overline{n}$: the average number of samples in each population, $C$: the coefficient of variation for the number of samples in different populations, $\overline{p}$:the average allele frequency for all populations, $s^{2}$: the variance of allele frequencies for different populations, $\hat{\theta}$: Weir & Cockerham's *F_ST_* unbiased estimate (Bhatia et al., 2013).

Bhatia, G., Patterson, N., Sankararaman, S., and Price, A. L. (2013). Estimating and interpreting FST: The impact of rare variants. *Genome Res.* 23, 1514–1521. doi: 10.1101/gr.154831.113

# 2 Supplementary Figures and Tables

## 2.1 Supplementary Figures

**Supplementary Figure 1** 3D-PCA explained distribution chart

**Supplementary Figure 2** ADMIXTURE genetic composition of *Phragmites australis* individuals across K = 2–19

**Supplementary Figure 3** Linkage disequilibrium decay plot. Note: The horizontal axis represents the distance between SNPs, and the vertical axis represents the *r²* value.

## 2.2 Supplementary Tables

**Supplementary** **Table 1** The details of 98 representative *Phragmites australis* samples from 10 geographic areas of Eastern China.

| SN | Symbol | Chinese Name | longitude | latitude | Description | Number of samples |
| --- | --- | --- | --- | --- | --- | --- |
| 1 | XZH | 锡祉湖 | 125.397 | 43.99796 | The lake inside the Northeast Institute of Geography and Ecological Environment, Chinese Academy of Sciences, located in ChangchunCity, Jilin Province | 8 |
| 2 | PJ | 盘锦 | 122.1553 | 40.63911 | A coastal city in Liaoning province | 10 |
| 8 | BYD | 白洋淀 | 116.0168 | 38.89023 | The largest freshwater lake in North China Plain, located in Baoding City, Hebei Province | 10 |
| 6 | DLJHK | 独流减河河口 | 117.5559 | 38.76767 | Duliujian River estuary, located in Tianjin municipality | 10 |
| 5 | HHK | 黄河口 | 119.2514 | 37.78198 | China's second largest river, the Yellow River estuary, has the famous Yellow River Delta, located in Dongying city, Shandong Province | 10 |
| 7 | WSH | 微山湖 | 116.8537 | 34.98807 | A Fault-depression lake in southern Weishan County, Jining City, Shandong Province, China. It is connected end to end with Zhaoyang Lake, Dushan Lake and Nanyang Lake, and is jointly called the South Four Lakes | 10 |
| 3 | CJK | 长江口 | 122.0008 | 31.30142 | The estuary of the Yangtze River, the largest river in Asia. Located in the northern part of Shanghai Municipality | 10 |
| 4 | MJK | 闽江口 | 119.6444 | 26.1351 | The estuary of the Minjiang River, the largest river flowing into the sea (East China Sea) in Fujian Province, China | 10 |
| 9 | LQW | 龙岐湾 | 114.4991 | 22.57654 | South Asia tropical monsoon climate, located in Shenzhen Dapeng New District Dapeng Peninsula coastal area | 10 |
| 10 | FCG | 防城港 | 108.3091 | 21.64487 | Located in the south of Guangxi Zhuang Autonomous Region and the southwesternmost part of the coastline of Chinese mainland | 10 |

­­SN: Serial number

**Supplementary** **Table 2** Results of comparison between sample and reference genome

| Sample ID | Mapped Ratio (%) | Proper Ratio (%) | Average Insert Size | Average Depth | Real Depth | Coverage (%) (≥1x) | Coverage (%) (≥4x) |
| --- | --- | --- | --- | --- | --- | --- | --- |
| LQW9 | 18.88 | 13.34 | 336.6 | 0.23 | 14.14 | 1.6 | 0.61 |
| LQW8 | 18.55 | 13.9 | 206.5 | 0.23 | 13.31 | 1.74 | 0.64 |
| LQW7 | 18.52 | 13.02 | 336.7 | 0.21 | 13.71 | 1.51 | 0.58 |
| LQW6 | 18.87 | 13.25 | 343.7 | 0.37 | 17.44 | 2.12 | 0.86 |
| LQW5 | 18 | 12.75 | 327.5 | 0.21 | 13.77 | 1.56 | 0.6 |
| LQW4 | 18.18 | 12.93 | 325.9 | 0.23 | 14.64 | 1.57 | 0.63 |
| LQW3 | 18.19 | 12.98 | 322 | 0.23 | 14.53 | 1.57 | 0.63 |
| LQW2 | 18.63 | 13.51 | 308.3 | 0.23 | 14.49 | 1.56 | 0.62 |
| LQW10 | 18.02 | 13.19 | 223.4 | 0.31 | 16.17 | 1.94 | 0.81 |
| LQW1 | 18.27 | 13.32 | 225.4 | 0.24 | 14.52 | 1.67 | 0.66 |
| WSH9 | 95.53 | 82.7 | 366.2 | 1.13 | 6.03 | 18.74 | 9.6 |
| WSH8 | 95.8 | 82.67 | 366.4 | 1.11 | 5.95 | 18.71 | 9.46 |
| WSH7 | 95.75 | 83.08 | 365.1 | 1.05 | 5.74 | 18.3 | 9.1 |
| WSH6 | 95.1 | 82.07 | 366.8 | 0.98 | 5.44 | 17.98 | 8.53 |
| WSH5 | 95.77 | 83.04 | 359.3 | 1.03 | 5.61 | 18.3 | 8.89 |
| WSH4 | 96.98 | 83.73 | 366.4 | 0.94 | 5.3 | 17.8 | 8.23 |
| WSH3 | 94.91 | 83.05 | 351.1 | 1.01 | 5.6 | 18.12 | 8.74 |
| WSH2 | 94.52 | 82.53 | 345.1 | 1.2 | 6.25 | 19.15 | 9.69 |
| WSH10 | 94.85 | 82.17 | 366.7 | 1.08 | 5.93 | 18.16 | 9.13 |
| WSH1 | 94.61 | 81.36 | 368.3 | 0.98 | 5.46 | 17.9 | 8.41 |
| PJ9 | 92.96 | 81.56 | 342.7 | 0.97 | 5.55 | 17.44 | 8.19 |
| PJ8 | 93.61 | 82.64 | 327.2 | 1.07 | 5.78 | 18.48 | 8.66 |
| PJ7 | 92.51 | 81.29 | 328.5 | 1.23 | 6.18 | 19.86 | 9.47 |
| PJ6 | 95.55 | 84.35 | 328.5 | 0.96 | 5.55 | 17.36 | 8.04 |
| PJ5 | 94.27 | 82.99 | 339.9 | 1.01 | 5.64 | 17.96 | 8.53 |
| PJ4 | 93.44 | 81.85 | 341.9 | 1.03 | 5.73 | 18.06 | 8.55 |
| PJ3 | 91.08 | 80.15 | 328.3 | 0.94 | 5.36 | 17.6 | 7.89 |
| PJ2 | 93.46 | 80.3 | 336.8 | 1.11 | 6.03 | 18.37 | 8.65 |
| PJ10 | 93.4 | 82.27 | 329.9 | 0.98 | 5.55 | 17.59 | 8.21 |
| PJ1 | 92.67 | 79.53 | 366.4 | 1.05 | 6 | 17.51 | 8.66 |
| MJK9 | 93.81 | 81.19 | 349.6 | 1.01 | 5.66 | 17.92 | 8.45 |
| MJK8 | 95.41 | 82.91 | 327.6 | 1 | 5.63 | 17.71 | 8.14 |
| MJK7 | 92.17 | 80.22 | 330.4 | 1.05 | 5.73 | 18.38 | 8.61 |
| MJK6 | 93.9 | 81.88 | 326 | 0.99 | 5.56 | 17.78 | 8.23 |
| MJK5 | 95.37 | 83.03 | 319.6 | 1.03 | 5.7 | 18.04 | 8.28 |
| MJK4 | 90.9 | 79.3 | 324.1 | 1.11 | 5.94 | 18.7 | 8.9 |
| MJK3 | 92.74 | 78.09 | 420.2 | 1.55 | 6.96 | 22.31 | 11.04 |
| MJK2 | 92.68 | 80.87 | 333.3 | 1.04 | 5.75 | 18.17 | 8.65 |
| MJK10 | 94.54 | 82.05 | 344.1 | 1.04 | 5.7 | 18.32 | 8.62 |
| MJK1 | 91.6 | 79.59 | 329.6 | 1.15 | 6 | 19.2 | 9.21 |
| HHK9 | 94.68 | 82 | 364.8 | 0.96 | 6.02 | 15.99 | 8.16 |
| HHK8 | 96.54 | 84.36 | 353.1 | 1.13 | 6.01 | 18.72 | 9.48 |
| HHK7 | 94.46 | 82.7 | 344.5 | 1.12 | 5.79 | 19.42 | 9.37 |
| HHK6 | 95.4 | 82.85 | 356.3 | 1 | 5.48 | 18.35 | 8.7 |
| HHK5 | 95.34 | 82.39 | 351.1 | 1.53 | 7.19 | 21.29 | 11.35 |
| HHK4 | 96.69 | 83.83 | 350 | 1.07 | 5.78 | 18.5 | 9.04 |
| HHK3 | 94.35 | 81.79 | 353.1 | 1 | 5.51 | 18.25 | 8.65 |
| HHK2 | 96.09 | 84.31 | 338.4 | 1.04 | 5.88 | 17.76 | 8.69 |
| HHK10 | 94.42 | 82.31 | 357.2 | 1.13 | 6.71 | 16.85 | 9.24 |
| HHK1 | 96.28 | 84.48 | 331.1 | 1.04 | 5.86 | 17.8 | 8.56 |
| FCG9 | 90.26 | 77.99 | 330.6 | 1 | 5.58 | 18 | 8.28 |
| FCG8 | 92.76 | 80.58 | 338.8 | 1.04 | 5.8 | 17.95 | 8.62 |
| FCG7 | 92.96 | 80.37 | 340.7 | 1.1 | 5.91 | 18.61 | 8.95 |
| FCG6 | 93.67 | 81.71 | 341.3 | 1.24 | 6.51 | 19.05 | 9.86 |
| FCG5 | 93.65 | 81.25 | 346.6 | 1.09 | 5.95 | 18.33 | 9 |
| FCG4 | 92.62 | 79.79 | 341.3 | 1.09 | 5.86 | 18.6 | 8.92 |
| FCG3 | 93.72 | 81.52 | 336 | 1.05 | 5.78 | 18.15 | 8.62 |
| FCG2 | 96.06 | 83.98 | 338.2 | 1.04 | 5.8 | 17.99 | 8.67 |
| FCG10 | 91.79 | 79.62 | 337.9 | 1.01 | 5.69 | 17.82 | 8.41 |
| FCG1 | 93.21 | 80.56 | 340.7 | 0.93 | 5.34 | 17.39 | 7.93 |
| DLJHK9 | 94.95 | 82.56 | 351.4 | 0.95 | 5.13 | 18.48 | 8.17 |
| DLJHK8 | 95.52 | 83.3 | 356.4 | 1.02 | 5.46 | 18.6 | 8.75 |
| DLJHK7 | 94.17 | 81.86 | 348.4 | 0.99 | 5.33 | 18.53 | 8.46 |
| DLJHK6 | 96.83 | 83.7 | 357.4 | 1.04 | 5.59 | 18.54 | 8.78 |
| DLJHK5 | 94.16 | 81.35 | 358.3 | 0.97 | 5.37 | 18.11 | 8.23 |
| DLJHK4 | 96.51 | 83.86 | 351.9 | 1.05 | 5.66 | 18.58 | 8.89 |
| DLJHK3 | 96.95 | 84.2 | 353.3 | 1.04 | 5.64 | 18.45 | 8.76 |
| DLJHK2 | 94.31 | 81.89 | 357.2 | 1 | 5.45 | 18.35 | 8.57 |
| DLJHK10 | 96.8 | 84.01 | 347.6 | 1.11 | 5.83 | 19.13 | 9.23 |
| DLJHK1 | 94.8 | 82.17 | 355.2 | 1.03 | 5.55 | 18.64 | 8.8 |
| CJK9 | 94.35 | 82.75 | 337.3 | 1.32 | 6.81 | 19.45 | 10.3 |
| CJK8 | 93.48 | 81.82 | 341.4 | 1.32 | 6.73 | 19.57 | 10.36 |
| CJK7 | 94.66 | 82.99 | 328.9 | 1.17 | 6.27 | 18.62 | 9.27 |
| CJK6 | 96.24 | 84.36 | 328.4 | 1.16 | 6.24 | 18.65 | 9.2 |
| CJK5 | 94.95 | 82.6 | 345 | 1.08 | 5.91 | 18.28 | 9.01 |
| CJK4 | 94.1 | 82.43 | 331.1 | 1.2 | 6.36 | 18.81 | 9.48 |
| CJK3 | 93.63 | 81.99 | 339 | 1.3 | 6.69 | 19.43 | 10.2 |
| CJK2 | 93.89 | 82.03 | 329.6 | 1.29 | 6.53 | 19.79 | 9.93 |
| CJK10 | 93.77 | 82.17 | 332.2 | 1.26 | 6.64 | 18.98 | 9.75 |
| CJK1 | 93.52 | 82.12 | 328.1 | 1.34 | 6.55 | 20.42 | 10.21 |
| XZH9 | 93.98 | 83 | 337.2 | 1.15 | 6.95 | 16.59 | 8.9 |
| XZH8 | 88.61 | 77.37 | 340.6 | 1.21 | 6.39 | 18.87 | 9.39 |
| XZH7 | 88.89 | 78.63 | 339.3 | 1.14 | 6.81 | 16.7 | 8.77 |
| XZH6 | 89.35 | 77.78 | 340.3 | 1.15 | 6.27 | 18.4 | 8.98 |
| XZH5 | 91.25 | 80.16 | 349.9 | 1.28 | 7.47 | 17.21 | 9.76 |
| XZH4 | 84.07 | 73.31 | 315.1 | 1.01 | 6.22 | 16.2 | 7.5 |
| XZH10 | 90.03 | 79.03 | 324.3 | 1.08 | 6.58 | 16.45 | 8.25 |
| XZH1 | 89.88 | 78.86 | 348.5 | 1.25 | 7.34 | 17.08 | 9.5 |
| BYD9 | 95.06 | 82.47 | 363.1 | 1.06 | 6.43 | 16.57 | 8.71 |
| BYD8 | 95.63 | 82.77 | 450.2 | 1.04 | 6.38 | 16.27 | 8.55 |
| BYD7 | 95.39 | 82.6 | 449.7 | 0.97 | 6.05 | 16.05 | 8.12 |
| BYD6 | 92.33 | 81.13 | 347.9 | 1.23 | 7.02 | 17.55 | 9.83 |
| BYD5 | 95.51 | 82.38 | 446.3 | 0.96 | 6.02 | 15.87 | 7.93 |
| BYD4 | 96.5 | 84.53 | 354.8 | 0.96 | 5.95 | 16.15 | 8.18 |
| BYD3 | 95.91 | 83.31 | 357.9 | 1.03 | 6.31 | 16.38 | 8.62 |
| BYD2 | 94.36 | 82.49 | 360.4 | 0.94 | 5.83 | 16.04 | 8.07 |
| BYD10 | 94.49 | 82.12 | 358.8 | 1.07 | 6.45 | 16.62 | 8.78 |
| BYD1 | 94.49 | 82.34 | 360.5 | 1.07 | 6.44 | 16.65 | 8.86 |

**Supplementary** **Table 3** The correspondence between kinship and kinship values

| kinship | kinship value |
| --- | --- |
| Duplication / Monozygotic twin | > 0.3536 |
| Parent offspring / Full sib | 0.1768- 0.3536 |
| 2nd degree relationship | 0.0884- 0.3536 |
| 3rd degree relationship | 0.0442- 0.0884 |
| Unrelated | < 0.0442 |

**Supplementary** **Table 4** Linkage Disequilibrium Analysis

| Pop id | half.decay | decay01 |
| --- | --- | --- |
| BYD | 1000 | 1000 |
| XZH | 1000 | 1000 |
| CJK | 964.178 | 1000 |
| DLJHK | 780.589 | 1000 |
| FCG | 1000 | 1000 |
| HHK | 1000 | 1000 |
| MJK | 807.167 | 1000 |
| PJ | 167.898 | 606.936 |
| WSH | 183.522 | 663.416 |
| LQW | 629.337 | 999.866 |

Note：

Pop ID：Population symbol;

half-decay (kb)：The decay distance when the LD coefficient drops to half of its maximum value;

decay01 (kb)：Attenuation distance when the LD coefficient drops to 0.1.
